# Improving Treatment Options for Patients with Double Refractory CLL

**DOI:** 10.3390/cancers17030430

**Published:** 2025-01-27

**Authors:** Ryan Jacobs, William Wierda

**Affiliations:** 1 Atrium Health Levine Cancer Institute, Wake Forest University School of Medicine, Charlotte, NC 28204, USA; 2 Department of Leukemia, The University of Texas MD Anderson Cancer Center, Houston, TX 77030, USA

**Keywords:** chronic lymphocytic leukemia, small lymphocytic lymphoma, Bruton’s tyrosine kinase, B-cell lymphoma-2 inhibitor, chimeric antigen receptor T-cell therapy, double refractory

## Abstract

The proliferation and survival of chronic lymphocytic leukemia (CLL) cells are heavily dependent on B-cell receptor (BCR) signaling and resistance to apoptosis. Approvals of multiple covalent Bruton’s tyrosine kinas inhibitors (cBTKis) as well as the B-cell lymphoma-2 inhibitor (BCL2i) venetoclax targeting these pathways have revolutionized the treatment of CLL and small lymphocytic lymphoma (SLL). The superiority of these treatments over chemoimmunotherapy has been proven in phase III studies in both treatment-naïve and relapsed refractory settings, leading to the majority of patients with CLL being treated sequentially with cBTKis and the BCL2i venetoclax as their first- and second-line therapies. While most patients with CLL respond for many years to these sequenced treatments, they are unfortunately not curative. There remains an unmet need for effective treatment options for patients who progressed after treatment with both cBTKis and BCL2i, also referred to as double refractory patients. Treatment options for double refractory CLL has improved recently with the approval of the non-covalent BTK inhibitor (ncBTKi) pirtobrutinib as well as the CD19 targeted chimeric antigen receptor T-cell (CAR T-cell) therapy lisocabtagene maraleucel (liso-cel). These recently approved treatment options for patients with CLL with at least two prior lines of therapy have fortunately demonstrated efficacy for double refractory CLL. Additionally, there are several novel treatment options in clinical development, including bi-specific antibodies, second-generation BCL2is, new ncBTKis, and BTK degraders. Understanding resistance mechanisms to existing cBTKis and venetoclax can potentially inform us of the best utilization of available treatment options for double refractory CLL and provide a personalized approach for these patients. In this review, a challenging example of a double refractory patient with CLL will serve as the basis for a review of available literature on the treatment of double refractory CLL/SLL.

## Simple Summary

High overall response rates with durable remissions have led to most patients with chronic lymphocytic leukemia being treated sequentially with covalent Bruton’s tyrosine kinase inhibitors and the B-cell lymphoma-2 inhibitor venetoclax as their first- and second-line therapies. Chronic lymphocytic leukemia remains a largely incurable disease, and double refractory patients will ultimately require additional treatment options. The non-covalent Bruton’s tyrosine kinase inhibitor pirtobrutinib, as well as the chimeric antigen receptor T-cell therapy lisocabtagene maraleucel, are currently available treatment options for double refractory patients with chronic lymphocytic leukemia. There are additional treatment options currently in clinical development to treat double refractory patients, including bi-specific antibodies, second-generation B-cell lymphoma-2 inhibitors, additional non-covalent Bruton’s tyrosine kinase inhibitors, and Bruton’s tyrosine kinase degraders. Understanding resistance mechanisms to existing treatments can offer insight into a personalized approach to the treatment of double refractory chronic lymphocytic leukemia.

## 1. Introduction

B-cell receptor (BCR) signaling is fundamental to development and differentiation of normal B cells, and the survival and proliferation of chronic lymphocytic leukemia (CLL) cells. BTK plays a crucial role in the BCR signaling pathway and the biology and clinical course of CLL and small lymphocytic lymphoma (SLL). In 2014, ibrutinib was approved by the US Food and Drug Association (FDA) as a first-in-class covalent BTK inhibitor (cBTKi) [1]. Initial approval of ibrutinib was in relapsed/refractory CLL and for previously untreated patients with chromosome 17 aberrations; in 2016, this approval was extended to previously untreated patients with CLL/SLL. Subsequently, second-generation cBTKis, acalabrutinib and Zanubrutinib, were approved that were more selective for BTK with less off-target kinase inhibition. In head-to-head comparisons in the relapsed setting, both acalabrutinib and zanubrutinib were shown to have superior safety over ibrutinib [2,3,4]. In the case of zanubrutinib, superior efficacy was also demonstrated with improved progression-free survival (PFS) relative to ibrutinib, which was maintained with extended follow-up [5].

B-cell lymphoma 2 (BCL2) overexpression leading to resistance to apoptosis represents an additional important target for the treatment of CLL/SLL. Venetoclax, a selective BCL2 inhibitor (BCL2i), combined with a CD20 monoclonal antibody, was shown to have excellent activity in the treatment of both previously untreated and relapsed/refractory CLL/SLL [6,7]. The development of cBTKis and venetoclax represents major advances in the treatment of patients with CLL/SLL, with head-to-head comparisons to chemoimmunotherapy showing improvements in PFS, and in some trials, overall survival (OS) [7,8,9,10]. National guidelines now favor the use of cBTKis or venetoclax in the treatment of patients with CLL/SLL over chemotherapy, and the use of chemotherapy is no longer considered a standard of care for treatment of CLL/SLL.

Notably, post hoc analysis of patients with CLL treated with ibrutinib in the first-line setting suggested an overall survival rate similar to an age-matched population without CLL [11]. Despite these remarkable breakthroughs in a relatively short period of time, as more and more patients progress on both cBTKis and venetoclax, additional treatments are needed to address this “double refractory” patient population. The patient case under discussion provides important examples of approaches that can maximize PFS before a patient develops double refractory CLL/SLL. These strategies include using an alternative cBTKi in the setting of prior intolerance as well as rechallenging with venetoclax after progression from fixed-duration venetoclax-based prior treatment. Beyond cBTKi and BCL2i treatment, patients with CLL/SLL now have access to the FDA approved non-covalent BTKi (ncBTKi) pirtobrutinib as well as the CD19 targeted chimeric antigen receptor T-cell (CAR T-cell) therapy lisocabtagene maraleucel (liso-cel). For the select patients who ultimately require treatment beyond these therapies available for double refractory patients, there are several novel treatment options under development in ongoing clinical trials including bi-specific antibodies, second-generation BCL2is, additional ncBTKis, and BTK degraders. It is foreseeable that in the future, we will sufficiently understand resistance mechanisms to existing cBTKis and venetoclax to inform treatment decisions for double refractory patients with CLL/SLL and provide a personalized approach for these patients.

## 2. Case Presentation

The following case details reflect a hypothetical case presentation based on actual clinical experience that is presented with the intention of guiding the subsequent detailed review of the approach to treatment in a patient with double refractory chronic lymphocytic leukemia (CLL). A 72-year-old female with relapsed/refractory CLL, originally diagnosed in 2007, presents for follow up with evidence of progressive lymphocytosis and worsening thrombocytopenia while being treated with acalabrutinib. The patient has had a long history of multiple treatments for her CLL, including bendamustine plus rituximab in 2012 followed by treatment with ibrutinib beginning in 2014 due to symptomatic relapse. Ibrutinib was ultimately discontinued in 2016 by the patient’s previous oncologist due to arthralgias without attempts at dose reduction. At the time of ibrutinib discontinuation, the patient had no clinical evidence of active disease, and she was followed off treatment. She ultimately progressed after 2 years of observation and presented to my clinic to discuss treatment options in the setting of symptomatic progression. She was the treated with venetoclax plus obinutuzumab in 2018 for a 2-year course of venetoclax that was completed in 2020. When she first progressed after completing the initial 2-year venetoclax course, the patient was subsequently rechallenged with single-agent venetoclax in 2022, but only transiently responded for approximately 10 months before ultimately progressing while on treatment. Given that the patient discontinued ibrutinib due to intolerance, not progression, she was started on acalabrutinib in late 2022. She responded to acalabrutinib for approximately 1 year and developed symptomatic CLL progression while on acalabrutinib. Throughout her multiple recurrences, repeat FISH analysis revealed no new FISH abnormalities, no clonal evolution, and the patient had a mutated IGHV gene and no *TP53* mutation on next-generation sequencing (NGS). An NGS panel revealed a T474I mutation in Bruton’s Tyrosine Kinase (BTK).

## 3. Dose Adjustments and Switching cBTKis in the Setting of Intolerance

It is important to recognize the notably long PFS associated with first-line cBTKi-based treatment. Long-term follow-up of the RESONATE-2 study, which investigated the efficacy of ibrutinib versus chlorambucil as first treatment for symptomatic patients with CLL, ultimately showed a median PFS of 8.9 years in the ibrutinib treated patients [12]. Extended follow-up showing similarly excellent first-line efficacy for both acalabrutinib and zanubrutinib was also reported [13,14]. Given the impressive activity of first-line cBTKis, attempting to mitigate toxicities through either dose reduction or switching to an alternative cBTKi before abandoning these effective treatment options may help in potentiating long-term disease control.

After first being treated with chemoimmunotherapy, our patient was later treated with ibrutinib for 2 years before discontinuing due to arthralgias. Many patients see their treatment-related toxicities improve or resolve on lower doses on ibrutinib, and can putatively achieve this without apparent negative impact on long-term treatment efficacy [15,16]. More time will be needed to follow-up on the long-term effects of dose adjustment’s impact on outcomes in acalabrutinib- and zanubrutinib-treated patients.

For patients who fail dose adjustment or for whom dose adjustment is not desired, there are available data to suggest that switching from one cBTKi to an alternative cBTKi will often lead to resolution of treatment-limiting toxicity and facilitate a patient’s ability to stay on cBTKi treatment. Awan et al. prospectively followed 33 patients that switched from ibrutinib to acalabrutinib due to toxicity. A total of 72% of these patients who switched saw no recurrence of the toxicity leading to ibrutinib intolerance, with an additional 13% of patients having the toxicity recur but at a lower grade. Only 14% of patients on this study saw no improvement in toxicity when switched to acalabrutinib [17]. A more recent report by Shadman et al. followed 67 patients that were followed prospectively after switching cBTKis to zanubrutinib due to toxicity to either ibrutinib or acalabrutinib. Sixty-eight percent of patients who switched from ibrutinib to zanubrutinib due to intolerance had their toxicity resolve. An additional 24% of these patients had toxicity recur, but at a lower grade, leaving only 8% of patients who had recurrence of the same toxicity from ibrutinib at a similar or higher grade after switching to zanubrutinib. A similar pattern was seen in patients who switched from acalabrutinib to zanubrutinib, with 73% of patients having their toxicity resolve with the switch to zanubrutinib and 11% had their toxicity recur but at a lower grade. Only 16% of the patients with CLL in the study who switched from acalabrutinib to zanubrutinib had recurrence of the same toxicity at a similar or higher grade [18].

In the case of our patient who was suffering from persistent arthralgias while on treatment with full-dose ibrutinib, the oncologist who was caring for her at the time opted for a treatment holiday as opposed to dose reduction. The patient self-referred to see me at the time of clinical progression of her CLL while on active surveillance. After discussing her options, she ultimately opted against initiating an alternative cBTKi and elected to proceed with venetoclax treatment. Eventually, when the patient required treatment after progressing on venetoclax, she was then treated with acalabrutinib, leading to a partial response which she maintained for approximately 1 year. She did not have recurrence of her arthralgias while on treatment with acalabrutinib.

## 4. Retreatment with Venetoclax

The CLL14 trial showed excellent long-term disease control in previously untreated symptomatic patients with CLL treated with 1 year of venetoclax and 6 months of obinutuzumab [6]. Long-term follow-up of the CLL14 trial showed an impressive median PFS of approximately 76 months with 1 year of this time defined treatment [19]. For relapsed/refractory patients with CLL, extended follow-up of the MURANO trial showed that patients treated with venetoclax for 2 years along with rituximab for the first 6 months had a median PFS of 53.6 months [20]. This is with the notable caveat that very few of the patients on the MURANO trial were previously treated with a cBTKi before receiving venetoclax-based treatment on MURANO. In the case under review, our patient was treated with venetoclax with CD20 mAb in the relapsed setting after having been previously treated with chemoimmunotherapy and ibrutinib; the latter of which was discontinued due to toxicity. After completing her 2-year course of venetoclax treatment, she went on observation and remained progression-free for close to 2 years before progressing with symptoms once again.

The efficacy of venetoclax-based retreatment was reported in a limited number of patients from the MURANO study. With 7 years of follow-up on the MURANO study, the PFS rate at 7 years for venetoclax-treated patients was 23%, and the median time to next treatment for these patients was 63 months. Thirty-seven percent of the venetoclax-treated patients had not received subsequent treatment for CLL after 7 years of follow-up, but of those who did require additional treatment, 25 patients ultimately received retreatment with venetoclax plus rituximab. The overall response rate (ORR) for retreatment with venetoclax plus rituximab was 72%, and the median PFS was 23.3 months [21]. Thompson et al. reported data from a multicenter international retrospective investigation reviewing outcomes for patients with CLL who were retreated with venetoclax after previous venetoclax treatment in a real-world setting. A total of 46 patients were identified, the overwhelming majority of whom had received their initial course of venetoclax in the relapsed setting (91%). Forty percent of these patients had received a cBTKi prior to receiving venetoclax. There was a nearly equal mix of patients who were retreated with venetoclax combined with CD20 mAb therapy versus venetoclax monotherapy retreatment, and there was a median of 16 months between the completion of their initial course of venetoclax and retreatment. The ORR to venetoclax retreatment was 80%, and the median PFS was 25 months. For the subgroup of patients with cBTKi exposure, the ORR and PFS was lower, with 56% responding to retreatment and a median PFS of 15 months [22].

In the CLL14 study, the limited number of patients who required treatment following clinically significant progression on the venetoclax plus obinutuzumab arm overwhelmingly received second-line therapy with a cBTKi, so the efficacy of retreatment with venetoclax following first-line venetoclax-based therapy remains undefined. Intuitively, less refractory patients with CLL with fewer prior lines of therapy who had only previously been treated with a 1-year course of venetoclax and obinutuzumab could potentially have higher ORR and PFS due to venetoclax rechallenge than was seen in the patients rechallenged with venetoclax in the relapsed setting reported from the MURANO trial. In order to explore the clinical benefit of venetoclax-based retreatment in patients previously treated with first-line venetoclax plus obinutuzumab, Davids et al. is conducting a prospective phase 2 study (ReVenG) investigating venetoclax-based rechallenge in patients who achieved at least a 12-month remission from their initial treatment with venetoclax. ORR and PFS will be assessed in enrolled patients who will be divided into two cohorts: cohort 1 will include patients progressing after more than 2 years following first-line treatment with venetoclax plus obinutuzumab, and cohort 2 will include patients who progressed between year 1 and year 2. Patients on this study will receive venetoclax retreatment along with 6 months of obinutuzumab. In cohort 1, patients will discontinue venetoclax after 12 months, and patients in cohort 2 will remain on venetoclax for a total of 24 months [23]. Results from this prospective study will provide more robust guidance on the efficacy of and approach to rechallenging patients with venetoclax, particularly as it pertains to the growing number of patients who were treated with venetoclax in the first-line setting.

Our heavily pretreated patient maintained her response to venetoclax retreatment for only 10 months before progressing while on therapy. Acquired mutations affecting the BH3-binding domain of BCL2 have been reported as a resistance mechanism to venetoclax, reducing the ability of venetoclax to bind to this domain [24]. In a small group of 15 patients, Blombery et al. reported that BCL2 G101V mutations were identified by NGS in 7 of the 15 patients who had progressed while on venetoclax [25]. In addition to G101V mutations, other pathways associated with resistance to venetoclax were reported, including overexpression of BCL-XL and MCL1 [26].

Given that our patient was previously intolerant, but not resistant to ibrutinib, he was next treated with acalabrutinib following progression on venetoclax. BTK sequencing for resistance mutations was performed prior to initiating acalabrutinib and there were no identified mutations. As noted previously, the patient initially responded to acalabrutinib treatment, but her response was transient and her CLL began to progress after approximately one year on treatment. At this point, the patient had disease that had progressed on both BCL2i and cBTKi, and her CLL would be appropriately deemed double refractory. BTK resistance testing was repeated on this patient at the time of progression on acalabrutinib and revealed a T474I BTK-resistant mutation.

## 5. BTK Resistance

Patients treated with cBTKi frequently achieve long-term remissions, but unfortunately, many develop resistance that ultimately drives progression of their CLL/SLL. The mechanisms of resistance to cBTKis have been extensively studied in patients progressing on ibrutinib [27,28]. The majority of patients who progress on ibrutinib develop mutations affecting the cysteine C481 (C481S being most common) residue in the kinase domain of BTK; additional resistant mutations have also been observed, including gain-of-function mutations at phospholipase C gamma 2 (PLCG2), a downstream signaling molecule of BTK [29,30]. Mutations at C481 interfere with the covalent binding of ibrutinib, acalabrutinib, and zanubrutinib, which share a similar mechanism of action. In a cohort of 112 patients at The Ohio State University progressing on ibrutinib whose disease was evaluated with NGS, Woyach et al. reported acquired mutations of BTK or PLCG2 in 87% of the patients. An eight-patient cohort in this group had clinical relapse while being followed prospectively with serial NGS testing, and all eight patients had BTK mutations at C481S with the expansion of the clone prior to relapse [31].

The data surrounding BTK resistance in patients treated with acalabrutinib and zanubrutinib is less extensive at this time relative to ibrutinib, with many questions remaining. In addition to C481 mutations, secondary resistance mechanisms specific to the newer cBTKis acalabrutinib and zanubrutinib have more recently been reported. C481 BTK mutations appear to also be the most commonly observed mutations in patients who progressed on acalabrutinib. Woyach et al. reported on 14 patients with CLL relapse on acalabrutinib whose samples were evaluated for full BTK and PLCG2 mutations using NGS. BTK mutations at C481S were found in 69% of the patients (9/16), while C481R and C481Y mutations were each seen in 1 patient, respectively. One patient was found to have a BTK T474I gatekeeper mutation, and two patients were found to have PLCG2 mutations that were coexisting in patients with C481S mutations [32]. Sun et al. performed NGS on the peripheral blood of 48 patients prospectively while on acalabrutinib. At the time of progression, 11/14 (79%) of the patients with progressive disease had newly acquired mutations. Six of the fourteen (43%) patients with detectable acquired mutations had mutations at C481S, and three of fourteen (21%) were found to have gatekeeper mutations at T474I [33].

BTK L528W mutation from leucine to tryptophan was reported as a common resistance mutation in patients progressing on zanubrutinib. Handunnetti et al. analyzed patients with CLL treated with zanubrutinib on four separate clinical trials across three centers who had serial samples available for full BTK mutation analysis. They identified four patients with CLL progression on zanubrutinib who underwent NGS of BTK and PLCG2, and all four were found to have L528W BTK mutations. Biochemical and cellular studies were performed on these patients, and the L528W BTK mutation resulted in significant loss of BTK activity compared to both BTK wild-type and patients with C481S mutation, leading to a resultant label of BTK L528W mutations as being “kinase dead” [34]. This report was later followed by Blombery et al. comparing patients who progressed on zanubrutinib to those progressing on ibrutinib. As a result, 7 of 13 patients progressing on zanubrutinib (54%) had L528W BTK mutations compared to only 1 of 24 patients progressing on ibrutinib (4%) (*p* = 0.001) [35]. Mutations at L528W have not been reported as significant drivers for resistance to ibrutinib or acalabrutinib. Recent data with a small number of patients suggest that L528W and/or T474I BTK mutations show cross-resistance with pirtobrutinib (Table 1) [35,36]. The presence of L528W or T474I mutations in patients with CLL progressing on zanubrutinib or acalabrutinib, respectively, has led to some concern that patients that progress with these mutations will ultimately not respond to ncBTKi-directed therapy with pirtobrutinib.

Interestingly, patients who progress early on treatment with cBTKi appear to possibly have differing drivers of resistance. Brown et al. reported BTK resistance data on the small number of patients who progressed early in treatment on the ALPINE trial, which compared the efficacy and tolerability of zanubrutinib versus ibrutinib for previously treated symptomatic patients with CLL/SLL. With a median follow-up of 17 months, 26 patients had progressed on zanubrutinib, and 31 patients had progressed on ibrutinib. Unexpectedly, neither BTK nor PLCG2 mutations were detected in the majority of patients on both arms of the study, with 81% of the patients progressing on zanubrutinib and 87% of the patients progressing on ibrutinib having no identifiable drivers of progression on NGS. On the ibrutinib arm, three patients had a C481S BTK mutation identified, and one patient had an isolated PLCG2 mutation. On the zanubrutinib arm, two of the five patients with recognized BTK mutations were mutated at L528W, and the remaining three of five patients had C481S BTK mutations [40]. Given that 83% (43/52) of the patients progressing early on cBTKi therapy on the ALPINE study had no detectable acquired BTK or PLCG2 mutations, it does not appear that mutations at BTK or PLCG2 are the primary factors driving progressive disease in patients who progress early on cBTKi treatment and that the primary driver of early resistance to cBTKi therapy remains undefined. It was the conclusion of Brown et al. that, given the low incidence of non-C481S mutations on the ALPINE study, that patients with CLL who were treated with zanubrutinib are likely to remain sensitive to ncBTKi-directed therapies.

## 6. Pirtobrutinib

As previously reviewed, most patients progressing on cBTKi will have a BTK-resistant mutation at C481S. Pirtobrutinib is an ncBTKi developed to maintain efficacy in these patients, with a mechanism of action defined by non-covalent reversible binding of BTK outside of the C481 binding site. In December 2023, the FDA approved pirtobrutinib for the treatment of relapsed/refractory CLL/SLL based on the results of the phase 1/2 BRUIN study. In this study, there were 247 relapsed patients with CLL/SLL who were previously treated with a cBTKi who received pirtobrutinib. The median number of prior lines of therapy in this patient population was 3 (ranging from 1–11), and 41% were also treated with the BCL2 inhibitor venetoclax. When including partial responses with lymphocytosis, the ORR was 82% with a median PFS of 19.6 months [41]. Importantly, pirtobrutinib was active in patients with BTK mutations at C481S [42,43]. Adverse events associated with the cBTKi were reported at relatively lower rates for pirtobrutinib, with 14% of patients experiencing hypertension, 4% experiencing atrial fibrillation/flutter, and 2% reported as having major hemorrhage. Remarkably, only 3% of patients discontinued pirtobrutinib due to a treatment-related adverse event [41]. Patients in this study were not identified based on whether they were refractory to prior treatment with cBTKis and BCL2i treatment, and patients could be enrolled in the BRUIN study with prior exposure to these treatments regardless of whether they had progressed on them or had discontinued treatment for reasons other than disease progression. When comparing patients on the BRUIN trial who had been exposed to both cBTKis as well as BCL2i treatment (i.e., “double exposed” patients), the ORR was similar to the overall BRUIN patient cohort (82% versus 79% for the double-exposed patients), but the PFS appeared to be reduced (19.6 months versus 16.8 months for the double-exposed patients).

Extended follow-up for the BRUIN study by Woyach et al. reported additional outcomes for the double-exposed patient population and compared 154 patients who had not received prior BCL2i therapy versus 128 patients who had been previously treated with venetoclax before receiving pirtobrutinib. With this extended follow-up, the differences in PFS between these two groups appeared more substantial, with a median PFS of 23 months among patients who were cBTKI-exposed but BCL2i-naïve versus 15.9 months for patients who had been exposed to prior treatment with both cBTKi and venetoclax [44].

Wang et al. published an early report on emerging mechanisms of resistance to pirtobrutinib. Of 55 treated patients, 9 patients with relapsed/refractory CLL had acquired mechanisms of genetic resistance to pirtobrutinib that were characterized. These included mutations at V416L, A428D, M437R, T474I, and L528W that were clustered in the kinase domain of BTK and that conferred resistance to pirtobrutinib. Mutations in BTK or PLCG2 were found in all nine patients who were progressing, and most of these mutations were also associated with resistance to the available cBTKis (Table 1). Transcriptional activation reflecting BCR signaling persisted in these progressing patients despite continued therapy with pirtobrutinib [36]. Brown et al. subsequently reported on the genomic evolution and resistance during pirtobrutinib therapy in cBTKi-pretreated patients with CLL in an updated analysis from the BRUIN study. Data were available for 86 patients with CLL enrolled on the BRUIN study who were pretreated with cBTKi and had paired NGS data available at baseline and at the time of progression on pirtobrutinib. Prior cBTKi use in these patients was dominated by ibrutinib (n = 77, 90%), followed by acalabrutinib (n = 15, 17%), and zanubrutinib (n = 2, 2%). At baseline, 46 (53%) of the patients harbored BTK mutations, predominantly C481S mutation in 53% (N = 45); however, 7% (N = 6) patients had a BTK T474I mutation. The variant allele frequency (VAF) of the BTK C481S mutations decreased or was completely cleared in the majority of patients (decrease in 86%, 36/42, complete clearance = 55%, 23/42) while on treatment with pirtobrutinib. At the point of disease progression on pirtobrutinib, 69% (59/88) of patients acquired at least 1 mutation, the majority of which were BTK mutations (44%, 39/88). Of the BTK mutations, the most common mutation detected was the gatekeeper mutation T474I (64% of BTK mutations, N = 25/39) or kinase-dead mutations with L528W (36% of BTK mutations,14/39). Six patients showed persistent BTK C481S mutation at the time of progression. Thirty-two percent (28/88) of patients who progressed on pirtobrutinib had no identifiable mutation on NGS, illustrating that the driver for a substantial proportion of patients progressing on pirtobrutinib remains unknown [45].

At the time of progression on acalabrutinib, our patient had acquired the gatekeeper BTK T474I mutation. The potential for resistance to pirtobrutinib with this mutation was discussed with the patient, and clinical trial with a MALT-1 inhibitor ABBV-525 was offered. The patient was a screen failure for the clinical trial due to baseline reduced GFR. The patient was subsequently treated with pirtobrutinib, but unfortunately was refractory to treatment and continued to have uninterrupted disease progression with worsening lymphocytosis and lymphadenopathy while on treatment. CAR T-cell therapy with liso-cel was discussed as a potential next option.

## 7. Lisocabtagene Maraleucel

Although the first experimental use of CAR-T cells to treat CLL was reported in 2011, further development for the treatment of relapsed/refractory CLL/SLL was outpaced by CAR T-cell therapy development in other hematological malignancies [46,47,48]. Lisocabtagene maraleucel (Liso-cel) is an autologous CD19-targeting CAR T-cell with a 4-1BB co-stimulatory domain and the final product containing CD4:CD8 CAR T-cells in a 1:1 ratio. Liso-cel was approved by the FDA in March 2024 for third-line or later treatment of patients with CLL/SLL, whose prior treatments included a BTKi and a BCL2i. The approval was based on the phase 1/2 TRANSCEND CLL–004 study, where Siddiqi et al. reported on the safety and efficacy in 87 patients the relapse/refractory CLL/SLL who received liso-cel after prior lymphodepleting chemotherapy. In the 49-patient double refractory cohort that had prior progression on cBTKi and BCL2i, there was and ORR of 43%, with 63% of patients obtaining undetectable minimal residual disease (uMRD) in blood, and 59% obtaining uMRD in the bone marrow. Complete remission (CR) was seen in only 9/49 of these patients (18%), however [49]. Extended 24-month follow-up was subsequently reported, and median PFS was 26.2 months for patients who achieved uMRD in blood analyses and only 2.8 months in patients with detectable MRD. The patients who achieved CR appear to have the most durable responses, with no patients progressing who achieved a CR with a median follow-up of 19.7 months in this select group [50].

Among all patients treated with liso-cel in the TRANSCEND CLL 004 study, grade 3 cytokine release syndrome was reported in 10 of 117 (9%) patients, and there were no grade 4 or 5 events. Grade 3 neurological events were reported in 21 (18%) patients with only one (1%) grade 4 and no grade 5 events. Only one death was felt to be related to liso-cel in the study, and this death occurred after the development of macrophage activation syndrome–hemophagocytic lymphohistiocytosis [50].

It appears that cBTKi treatment with ibrutinib has beneficial effects on the tumor microenvironment and can expand CAR T-cells. Combining cBTKi treatment with CAR T-cell therapy may be a strategy that can overcome the resistance of CLL to CD19-targeted CAR T-cell therapies and improve responses [51]. In the phase 1/2 TRANSCEND CLL 004 trial, patients treated with combined ibrutinib and liso-cel showed an ORR of 95% in 19 relapsed/refractory patients with CLL, all of whom had previously received ibrutinib as a prior treatment for their CLL [52]. Our patient was offered treatment with liso-cel, but after discussing the schedule for treatment as well as the available data from the TRANSCEND CLL 004 study, she ultimately chose to pursue enrollment on a clinical trial with and alternative ncBTKi, LP–168.

## 8. Novel Non-Covalent BTK Inhibitors

There are other ncBTKis under clinical development that may provide alternative options to pirtobrutinib for patients with CLL/SLL. Nemtabrutinib (formerly ARQ-531) is an ncBTKi with available data suggesting it has high potency against both wild-type and C481S-mutated BTK. In addition to non-covalent irreversible binding at BTK, nemtabrutinib targets other kinases in the BCR signaling pathway, thus also potentially providing a treatment option for patients with PLCG2 mutations [53]. In a recent analysis of the phase 1/2 BELLWAVE-001 trial, Woyach et al. reported on the activity of nemtabrutinib in 57 patients with relapsed/refractory CLL/SLL who were treated at the recommended phase 2 dose of 65 mg. A total of 54 patients (95%) had a prior BTKi, and 36 patients (63%) had a C481S mutation. With the median follow-up of 8.1 months, ORR was 56%, and median PFS was 26.3 months. In a subgroup analysis of 24 patients (42%) who had both prior BTKi and venetoclax exposure, the ORR was 58% and was not significantly different from the overall trial population, but the median PFS was shorter in this double-exposed patient population at 10.1 months. Adverse events led to the discontinuation of the therapy in only 8% of the patients [54]. The shorter PFS reported for patients who were previously treated with cBTKi and BCL2i mirrored a similar trend that was seen in the BRUIN trial with pirtobrutinib in double-exposed patients discussed previously.

Two additional ncBTKis have been evaluated in clinical trials: vecabrutinib and fenebrutinib. The study of vecabrutinib did not proceed to phase 2 because of the lack of clinical activity [55]. Development of fenebrutinib was also terminated for similar reasons, although it did show limited activity in select patients with CLL who harbored BTK C481S mutation [56]. More recently, Woyach et al. reported on the activity of LP-168, deemed a “fourth-generation BTKi” with dual covalent and non-covalent BTKi activity in patients with relapsed/refractory CLL. Thirty-three patients with relapsed/refractory CLL were evaluable for response; 94% of these patients had received prior cBTKi and 11% had also been treated with ncBTKi. The majority of the patients on the trial, 21/33 (64%), harbored BTK C481S mutations. Seven of the thirty-three patients (21%) had a gatekeeper mutation at BTK T474I, and five patients (15%) had PLCG2 mutations. With a median follow-up of 12.6 months, the ORR was 54.5%, all partial responders. Ten out of the fifteen patients (67%) who received 200 mg or higher doses of LP-168 responded and the subgroup of patients with a BTK T474 I mutation had an ORR of 75% (3/4). No DLTs were observed as of the data lock for this report [37].

## 9. BTK Degraders

An alternative BTK-targeted approach currently under investigation is to overcome the resistance to both cBTKis and ncBTKis by degrading the BTK protein itself. BTK degraders are proteolysis-targeting chimeras (PROTCACs) that function by inducing catalytic ubiquitination of BTK via recruitment of the cereblon E3 ubiquitin ligase complex [57,58]. This results in the eventual BTK degradation by the proteasome, which theoretically would not be vulnerable to the known mechanisms of resistance to cBTKis and ncBTKis [59]. Specifically, BTK degraders can bind to BTK proteins with known BTK resistance mutations (C481, T474I, etc.) [60]. NX-2127 is a PROTAC BTK degrader that also degrades IKAROS family zinc finger 1 (IKZF1) and IKZF3, inducing immunomodulatory activity [61]. Preliminary data of the phase 1 NX-2127-001 were reported with 17 relapsed/refractory patients with CLL who had received a prior BTKi and 13 patients (77%) had also received venetoclax. The reported mean in vivo BTK degradation was 83%, and the ORR increased with longer follow-up in the 12 patients who were evaluable (17% at 2 months, 43% at 4 months, and 50% at 6 months). Responses were also observed in double refractory patients and those who had progressed on an ncBTKi [62].

The BTK degrader NX-5948 is an oral small molecule that selectively degrades BTK. Preliminary data of the ongoing phase-1 NX-5948-301 trial showed rapid and sustained BTK degradation with NX-5948 [59]. Shah et al. presented on 30 relapsed/refractory patients with CLL treated with NX-5948 evaluable for response, with four median prior lines of treatment, 88% of which had been treated previously with both cBTKi and BCL2i. The ORR was 77%, and responses were seen in the double refractory patients as well as patients who had also been previously treated with pirtobrutinib. Responders included patients with cBTKi resistant mutation C481S and PLCG2 as well as ncBTKi resistance mutations L528W and T474I [38]. BGB-16673 is another BTK degrader currently under clinical development for B-cell malignancies including CLL. Thompson et al. recently reported on 49 patients with CLL treated with BGB-16673 and four median prior lines of therapy, including 86% who were previously treated with cBTKis and BCL2i. The ORR was 78%, and the ORR increased to 94% (15/16) for the patients treated at 200 mg dose. Like NX-5948, responders in double refractory patients as well as patients previously treated with pirtobrutinib were also seen in this study [39].

## 10. Phosphoinositide 3-Kinase Inhibitors

Other drugs were developed that inhibit downstream of the BCR signaling pathway, including phosphoinositide 3-Kinase Inhibitors (PI3Kis). Idelalisib, duvelisib, and umbralisib are all PI3Kis that were approved by the FDA for the treatment of relapsed/refractory CLL/SLL [63]. The reduced activity of PI3Kis in the relapsed/refractory CLL population relative to that of cBTKis and venetoclax historically led their use in later lines of therapy. Initial reports of PI3Ki activity showed ORRs of 72% and 74% and median PFS values of 15.8 months and 13.3 months for idelalisib and duvelisib, respectively [64,65]. The patient populations on these trials were not representative of the patients with CLL who were receiving PI3Kis in the community, as no patients in these studies had received treatment with a prior cBTKi or venetoclax. Idelalisib was compared prospectively to both acalabrutinib and pirtobrutinib in separate studies in patients with relapsed CLL and was found to be inferior and more toxic [9,66,67]. The activity of PI3Kis was evaluated in a real-world analysis of patients with double refractory CLL, with an ORR of 47% and median PFS of only 5 months [68].

In addition to inferior efficacy relative to cBTKis and BCL2i treatment, PI3Ki use has been hindered by severe adverse effects including hepatotoxicity, diarrhea and colitis, skin toxicity, and infections. Toxicities such as these have led to high rates of treatment discontinuation [63]. Umbralisib did show a greater selectivity towards δ isotypes of PI3K, which was clinically associated to less pronounced toxicity and better tolerability [63]. Umbralisib was evaluated in patients who discontinued treatment with cBTKis or an alternative PI3Ki and showed an ORR of 44% and median PFS of 23.5 months [69]. After initial approval for the treatment of relapsed/refractory CLL/SLL, umbralisib was later voluntarily withdrawn from the market due to growing concerns of infection-related adverse events on extended follow-up. Development of other agents targeting PI3K (zandelisib, parsaclisib) was ultimately halted due to similar concerns [63]. The PI3Kis idelalisib and duvelisib remain available, and strategies for intermittent dosing of duvelisib have shown success in mitigating toxicity without reducing efficacy [70]. PI3Kis might be a possible treatment option for select patients, although the PFS in a double refractory setting would likely be short.

## 11. Novel BCL2 Targeted Therapies

There are currently four different BTKis approved for the treatment of CLL/SLL, and there are multiple additional BTKi options discussed above that will likely become available soon. Similarly, novel BCL2is such as sonrotoclax, lisaftoclax, and ABBV-453 are currently under development. While there are some preliminary data for relapsed/refractory CLL with these agents, the clinical data among patients who progressed on venetoclax who were later treated with a second-generation BCL2i remains very limited. Lisaftoclax is a highly selective and potent BCL2i, and data from 141 relapsed/refractory patients with CLL/SLL in a phase 2 trial was reported. Only a small number of patients on this trial were heavily pretreated with novel agents [15 (11%) had progressed on a cBTKi and only 3 (2%) had progressed on venetoclax]. The ORR with lisaftoclax monotherapy was 65% in this relapsed patient population [71]. Zhou et al. presented updated efficacy with lisaftoclax in relapsed patients with CLL across two clinical trials. No patients were reported as having been previously treated with venetoclax, and only 23% of the patients received a prior cBTKi. With a median follow-up of 14 months, 47 patients had completed ramp-up and had an ORR of 73% with a 24% CR rate. uMRD was achieved in 39% of patients tested in blood and the 24-month PFS was 39% [72].

Sonrotoclax is another novel BCL2i with selectivity to BCL2 and a potent pharmacokinetic profile that shows promise of being more selective than venetoclax and potentially effective against common mechanisms of BCL2i resistance [73,74]. According to the preliminary data in the phase-1 BGB-11417-101 trial, the ORR with sonrotoclax monotherapy was 67% in heavily pretreated patient population (6/8) [75]. Sonrotocolax is currently being developed combined with zanubrutinib for a combined 1 year of treatment as an all-oral first-line treatment for symptomatic patients with CLL [76].

## 12. Bispecific Antibodies

Bispecific antibodies (bsAbs) are an immunotherapeutic approach bringing T-cells into close proximity to tumor cells, leading to potent anti-tumor activity. CD3/CD20 bsAbs were reported to have significant activity in treating several hematological malignancies, and there are multiple bsAbs approved in the treatment of diffuse large B-cell lymphoma and follicular lymphoma [77].

Epcoritamab is a subcutaneously administered CD3/CD20 bsAb, and its activity in treating patients with relapsed/refractory CLL/SLL was investigated in the phase 1b/2 EPCORE CLL-1 trial. In this trial, epcoritamab was given as a monotherapy and also combined with venetoclax [78]. Updated results from Danilov et al. reported data in 40 patients with a median of four prior lines of therapy that were treated with single-agent epcoritamab. All patients had received prior cBTKi, and the majority were previously treated with both cBTKi and BCL2i (85%). The ORR was 61% with a 39% CR rate, and specifically, ORR was 53% and the CR rate 37% in the double-exposed patient population (n = 19). Among all patients, the median PFS was 12.8 months, and the median OS was not reached. Seventy-five percent of the responders who were evaluable for MRD achieved uMRD in the blood. Immune-mediated toxicities were manageable, with no grade 3+ CRS reported in the optimization cohort that had applied an additional step-up dose. No ICANS events were reported in this optimization cohort [79].

## 13. Allogenic Hematopoietic Stem Cell Transplantation

With the impressive efficacy of the growing number of treatment options available for patients with CLL/SLL, the use of allogenic hematopoietic stem cell transplantation (alloHCT) in the treatment of relapsed/refractory patients with CLL has been declining. AlloHCT remains an option in select refractory patients in whom approved options have been exhausted and clinical trial enrollment is not a possibility. AlloHCT has previously been shown to be effective in double refractory patients. Roeker et al. investigated the efficacy of alloHCT in a retrospective cohort study that identified 65 patients with relapsed/refractory CLL/SLL who had been previously treated with at least one small molecule inhibitor, including cBTKi, BCL2i, or PI3Ki. At 2 years, PFS was 63%, OS 81%, and the incidence of relapse was only 27%. AlloHCT was relatively safe with a non-relapse mortality of only 13%. The cumulative incidence of grade 3/4 acute graft-versus-host disease at day 100 was 24%, and moderate/severe GVHD developed ultimately in 27% of patients. Outcomes did not appear to be substantially worse in the patients who were double refractory versus those who had only progressed on one prior novel agent [80]. An additional retrospective analysis of 108 patients who received alloHCT and had been previously exposed to targeted agents also showed great efficacy. In this heavily pretreated patient population (median prior therapies = 4), a 3-year OS of 87% and 3-year PFS of 69% were found [81].

Like most patients with multiply relapsed CLL, our 72-year-old patient was advanced in age with multiple medical comorbidities when she became double refractory. At the point where the patient eventually was found to be refractory to treatment with pirtobrutinib, she was not deemed a good candidate for alloHCT. Further, with the availability of CD19-CAR T-cell therapy with liso-cel and its low incidence of treatment-related morbidity and mortality relative to alloHCT, particularly in an older patient population, strong consideration should be given to first utilizing CAR T-cell therapy prior to alloHCT in the select patients who are eligible for both treatment modalities.

## 14. Conclusions

**Figure 1:**
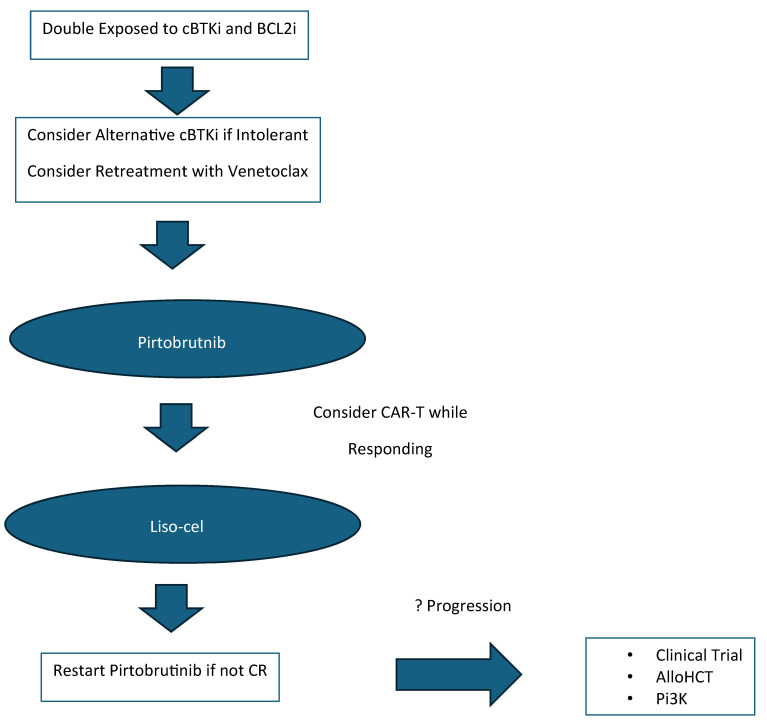
Approach to double refractory patients.

Small molecule inhibitors targeting key proteins in the BCR signaling pathway as well as the BCL2-mediated apoptotic pathway have significantly improved clinical outcomes of patients with CLL/SLL. The landscape of CLL/SLL treatment has forever changed after the introduction of the cBTKis ibrutinib, acalabrutinib, and zanubrutinib as well as the BCL2i venetoclax. With the availability of cBTKis and BCL2i therapy, most patients with CLL/SLL should realistically expect long-term disease control and, potentially, normalization of life expectancy. As more and more patients are treated with these small molecule inhibitors, there will be a growing number of patients who become double refractory. There is a growing body of research revealing the mechanisms that underlie resistance to cBTKis and BCL2i treatment with venetoclax. Drug development based on this research is leading to a growing number of novel targeted agents, including ncBTKis, BTK degraders, second-generation BCL2is, bispecific antibodies, and CAR T-cell therapies. New targets remain potential therapeutic opportunities, such as MCL-1, MALT-1, and ROR-1 as well. The recent approval of pirtobrutinib provides us an available ncBTKi treatment option and is now considered a standard-of-care treatment for double refractory disease. Expectations for responses to pirtobrutinib in the double refractory setting unfortunately appear to be relatively short for most patients, but the availability of CAR-T therapy with liso-cel has expanded the treatment armamentarium for patients with relapsed/refractory CLL/SLL as well (Figure 1). Future combination strategies with immunotherapies and targeted therapies are being extensively investigated as possible routes to reducing resistance to treatment. Thankfully, there are a growing number of potential new treatment strategies for double refractory disease with currently approved agents and available clinical trials.

From the point that our patient under review was double refractory, she failed to respond to pirtobrutinib and ultimately declined liso-cel and pursued treatment with a fourth-generation BTK inhibitor LP-168 on a clinical trial. At the time of writing this review, the patient is currently responding to treatment with normalization of lymphocytosis and resolution of thrombocytopenia.

## Figures and Tables

**Table 1:** Binding affinities and clinical efficacy of available BTKi to known mutations, relative to wild-type BTK [36,37,38,39].

		Wild-Type	C481S	T474I	L528W	A428D
Covalent BTKi	Ibrutinib	Normal	Decreased	Normal	Absent	Absent
	Acalabrutinib	Normal	Decreased	Decreased	Decreased	Absent
	Zanubrutinib	Normal	Decreased	Decreased	Absent	Absent
Non-covalent BTKi	Pirtobrutinib	Normal	Normal	Decreased	Absent	Absent
Dual covalent + noncovalent BTKi	LP-168	Normal	Normal	Normal	Absent	Absent
BTK Degraders	BGB-16673 NX-5948	Normal	Normal	Normal	Normal	Normal
